# Changes at glutamate tripartite synapses in the prefrontal cortex of a new animal model of resilience/vulnerability to acute stress

**DOI:** 10.1038/s41398-023-02366-w

**Published:** 2023-02-18

**Authors:** Tiziana Bonifacino, Jessica Mingardi, Roberta Facchinetti, Nathalie Sala, Giulia Frumento, Elona Ndoj, Marta Valenza, Caterina Paoli, Alessandro Ieraci, Carola Torazza, Matilde Balbi, Michele Guerinoni, Nadeem Muhammad, Isabella Russo, Marco Milanese, Caterina Scuderi, Alessandro Barbon, Luca Steardo, Giambattista Bonanno, Maurizio Popoli, Laura Musazzi

**Affiliations:** 1 Department of Pharmacy, Unit of Pharmacology and Toxicology, University of Genoa, Genoa, Italy; 2 School of Medicine and Surgery, University of Milano-Bicocca, Monza, Italy; 3 Department of Molecular and Translational Medicine, University of Brescia, Brescia, Italy; 4 Department of Physiology and Pharmacology “Vittorio Erspamer”, SAPIENZA University of Rome, Rome, Italy; 5 Laboratory of Neuropsychopharmacology and Functional Neurogenomics, Dipartimento di Scienze Farmaceutiche, Università Degli Studi di Milano, Milano, Italy; 6 Pharmacology Unit, School of Pharmacy, University of Camerino, Camerino, Italy; 7 Department of Theoretical and Applied Sciences, eCampus University, Novedrate, Como, Italy; 8 Genetics Unit, IRCCS Istituto Centro S. Giovanni di Dio, Fatebenefratelli, 25125 Brescia, Italy; 9 IRCCS Ospedale Policlinico San Martino, Genoa, Italy

**Keywords:** Molecular neuroscience, Depression

## Abstract

Stress represents a main risk factor for psychiatric disorders. Whereas it is known that even a single trauma may induce psychiatric disorders in humans, the mechanisms of vulnerability to acute stressors have been little investigated. In this study, we generated a new animal model of resilience/vulnerability to acute footshock (FS) stress in rats and analyzed early functional, molecular, and morphological determinants of stress vulnerability at tripartite glutamate synapses in the prefrontal cortex (PFC). We found that adult male rats subjected to FS can be deemed resilient (FS-R) or vulnerable (FS-V), based on their anhedonic phenotype 24 h after stress exposure, and that these two populations are phenotypically distinguishable up to two weeks afterwards. Basal presynaptic glutamate release was increased in the PFC of FS-V rats, while depolarization-evoked glutamate release and synapsin I phosphorylation at Ser^9^ were increased in both FS-R and FS-V. In FS-R and FS-V rats the synaptic expression of GluN2A and apical dendritic length of prelimbic PFC layers II–III pyramidal neurons were decreased, while BDNF expression was selectively reduced in FS-V. Depolarization-evoked (carrier-mediated) glutamate release from astroglia perisynaptic processes (gliosomes) was selectively increased in the PFC of FS-V rats, while GLT1 and xCt levels were higher and GS expression reduced in purified PFC gliosomes from FS-R. Overall, we show for the first time that the application of the sucrose intake test to rats exposed to acute FS led to the generation of a novel animal model of resilience/vulnerability to acute stress, which we used to identify early determinants of maladaptive response related to behavioral vulnerability to stress.

## Introduction

Stress is considered a primary risk factor for neuropsychiatric disorders [1, 2]. Accordingly, many animal models of psychopathology are largely based on exposure to validated chronic stress protocols. However, a number of studies have investigated the long-term consequences of acute stress, finding structural and functional changes that often resemble those induced by chronic stress [3–9]. Using a standard protocol of acute inescapable footshock (FS) stress [10, 11], we have previously shown that traumatic stress can induce both rapid and long-lasting structural and functional alterations in rat prefrontal cortex (PFC). The changes include: (a) corticosterone-dependent rapid enhancement of glutamate release and excitatory synaptic transmission, accounted for by increased trafficking of glutamate synaptic vesicles into the readily releasable pool (RRP), in turn mediated by phosphorylation of synapsin I at Ser^9^, and sustained for at least 24 h [10, 12, 13]; (b) a remarkable increase of the total number of excitatory nonperforated and axoshaft synapses in prelimbic PFC in only 40 min [8, 14]; (c) a shortening and simplification of apical dendrites in prelimbic PFC layers II–III pyramidal neurons, which were measurable already after 24 h and sustained for up to 2 weeks after stress [8, 15]; (d) a rapid increase of synaptic energy metabolism [16]; (e) an impairment in working memory and induction of anhedonic behavior, anxious phenotype, and fear conditioning [15, 16]. Nevertheless, in the previous studies, we have considered stressed animals as one single population, while individual animals may respond differently to stress exposure, as consistently reported for several chronic stress models [17–19] and only recently demonstrated also after acute stress [9]. Accordingly, stressed animals can be distinguished as resilient and vulnerable based on different behavioral, cellular, and molecular changes in the long-term stress response. Importantly, the study of the acute stress response may identify critical early determinants of maladaptive trajectories diverging from the pro-adaptive (resilient) physiological stress response [2, 20, 21].

Astrocytes have increasingly gained attention as major players in shaping neuronal synaptic function, strength, and plasticity, thus regulating brain functions [22]. Indeed, astrocytes do not only support neurons in maintaining optimal neuronal activity, but also dynamically interact with synapses modulating information processing [23–25] in a well-structured anatomic and functional assembly formed by perisynaptic astrocytes, presynaptic terminals, and the post-synaptic moiety, leading to the widely accepted concept of a tripartite synapse [26–28].

Alterations of astrocytic function have been implicated in mental disorders [29–31], and in the maladaptive response to acute stress [32, 33].

In the present study, we introduce a new rodent stress model based on acute inescapable FS stress, which may identify early determinants of resilient vs. vulnerable trajectories of the stress response [2, 20]. The rats were deemed resilient/vulnerable based on their behavior in the sucrose intake test for anhedonia performed 24 h after acute stress. Synaptic and perisynaptic structural/functional alterations at PFC glutamatergic synapses were measured in resilient/vulnerable rats 24 h after stress. We report distinct changes in resilient/vulnerable rats for synaptic and perisynaptic glutamate release, glutamate receptors, dendritic development, neurotrophins, and astrocytic proteins.

## Materials and methods

Detailed information is reported in the Supplementary Material.

### Animals

All experimental procedures involving animals were performed following the European Community Council Directive 2010/63/UE and were approved by the Italian legislation on animal experimentation (Decreto Legislativo 26/2014, animal experimentation licenses N 521/2015-PR and 140/2014-B—DGSAF24898). Experiments were performed with male Sprague-Dawley rats (175–200 g in weight at the beginning of the protocol, 350–450 g at the end). All the animals were sacrificed by beheading.

### Sucrose intake test and classification of resilient and vulnerable animals

**Fig. 1:**
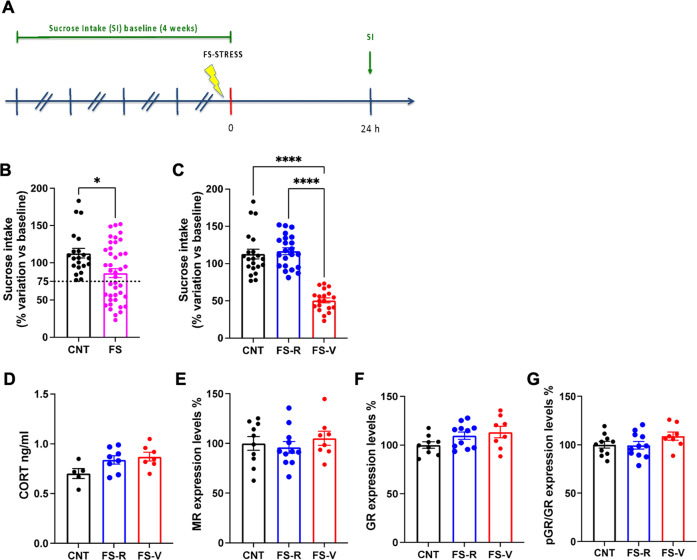
Generation of a rat model of resilience and vulnerability to acute footshock stress. **A** Experimental plan timeline. Basal (spontaneous) sucrose intake was established exposing the rats to the sucrose intake test twice a week for 4 weeks. Animals were then subjected to a single session of FS and sucrose intake test was performed again 24 h after FS. **B** Sucrose intake measured 24 h after FS. Mann Whitney test, *p < 0.05; *N* = 21 (CNT), 41 (FS). **C** Sucrose intake measured 24 h after FS with separation of resilient (FS-R) and vulnerable (FS-V) rats applying a 75% of sucrose intake cut-off. Kruskal-Wallis test, Dunn’s post-hoc test: ****p < 0.0001; *N* = 21 (CNT), 22 (FS-R), 19 (FS-V). **D** Corticosterone (CORT) serum levels. One-way ANOVA; *N* = 5 (CNT), 8 (FS-R), 7 (FS-V). **E** Mineralocorticoid receptor (MR) protein expression levels in PFC nuclear fraction. One-way ANOVA; *N* = 10 (CNT), 11 (FS-R), 8 (FS-V). **F** Glucocorticoid receptor (GR) protein expression levels in PFC nuclear fraction. One-way ANOVA; *N* = 9 (CNT), 11 (FS-R), 8 (FS-V). **G** phospho-Ser^203^-GR/GR protein expression levels in PFC nuclear fraction. One-way ANOVA; *N* = 10 (CNT), 11 (FS-R), 8 (FS-V).

Sucrose intake was evaluated as in [15], twice a week for 4 weeks. The average sucrose solution volume drunk by each animal was calculated and defined as baseline sucrose intake. After 4 weeks, animals were randomly assigned to FS (see below) or left undisturbed in their home cages (control). The sucrose intake test was repeated the day after, and the percent sucrose intake vs. baseline was calculated for each animal (Fig. 1A). Animals showing at least a 25% within-subject decrease in sucrose intake were considered anhedonic and classified as vulnerable (FS-V); all the others were defined as resilient (FS-R).

### Footshock stress procedure

Animals were subjected to a single session of acute inescapable FS stress as previously reported [15].

### Serum corticosterone assay

Blood Serum corticosterone levels were measured using a commercial kit (Corticosterone EIA kit, Enzo Life Sciences Inc., Farmingdale, NY) as previously reported [16].

### Preparation of subcellular fractions

Immediately after sacrifice, the PFC was rapidly collected in ice, homogenized in 10 volumes of Tris-buffered 0.32 M sucrose, and centrifuged at 1000 g for 5 min to obtain the nuclear fraction [34]. Purified synaptic terminals (synaptosomes) and glial perisynaptic processes (gliosomes) were purified on a discontinuous Percoll^®^ gradients in Tris-buffered 0.32 M sucrose as previously described [15, 35–37]. Synaptic membranes were prepared as in [12].

### Measurement of neurotransmitter release from purified synaptosomes and gliosomes

Synaptosomes and gliosomes were labeled with 0.05 µM [^3^H]D-Aspartate ([^3^H]D-Asp; a non-metabolizable analogous of glutamate used to label glutamate releasing pools; [38, 39]). Aliquots were distributed on microporous filters placed at the bottom of a set of 24 parallel superfusion chambers maintained at 37 °C (Superfusion System, Ugo Basile, Comerio, Varese, Italy) and processed as previously described [36, 40, 41].

### Western blotting

Protein concentration was calculated by Bradford or BCA assays (Sigma-Aldrich, Milano, Italy and Thermo Fisher Scientific, Milano, Italy, respectively) and 10–30 micrograms were loaded on acrylamide SDS-PAGE gels. Western blotting was performed as previously described [42, 43].

### Golgi-Cox staining and dendritic analysis

Golgi-Cox staining was performed using the Rapid Golgi Stain Kit (FD NeuroTechnologies, Inc., Columbia, MD, United States) on a dedicated set of animals as previously reported [15]. Dendritic length and branching, and Sholl analysis of pyramidal neurons within prelimbic PFC layers II–III were evaluated.

### Real time-quantitative polymerase chain reaction (RT-qPCR)

RT-qPCR was performed as previously described [44, 45]. Primer sequences used were: Bdnf: forward primer (fwd) 5′- GGGACTCTGGAGAGCGTGAA - 3′; reverse primer (rev) 5′- GTCAGACCTCTCGAACCTGC - 3′; Gdnf: fwd 5′- CACCAGATAAACAAGCGGCG - 3′; rev 5′- TCGTAGCCCAAACCCAAGTC - 3′; Hprt: fwd 5′- TCCCAGCGTCGTGATTAGTGA - 3′; rev 5′- CCTTCATGACATCTCGAGCAAG - 3′; Tbp: fwd 5′- TGGGATTGTACCACAGCTCCA - 3′; rev 5′- CTCATGATGACTGCAGCAAACC - 3′.

### Statistical analysis

Statistical data analysis was carried out using GraphPad Prism 9 (GraphPad Software Inc., USA). Results are presented as means ± standard error of the mean (SEM).

Normal distribution was verified using Kolmogorov–Smirnov test. For normally distributed data, statistical analyses were performed with unpaired Student’s *t*-test, one or two-way analysis of variance (ANOVA), and mixed-effects model when appropriate, followed by post-hoc multiple comparison tests as indicated in the figure legends. For non-normally distributed data, statistical analyses were performed with the Mann Whitney test (when 2 groups were compared) or Kruskal-Wallis test, followed by Dunn’s multiple comparison test.

The number of animals used in each experiment is indicated in the figure legends.

## Results

### Acute footshock stress induces anhedonic behavior in vulnerable animals that is preserved for up to two weeks

As previously reported [15], we found a significant reduction of sucrose intake in stressed animals (Mann Whitney test, *p* < 0.05), suggesting anhedonic behavior (Fig. 1B). However, looking at the distribution of single values, we observed that not all the rats displayed a reduction of sucrose intake compared to pre-stress condition. Thus, setting up an arbitrary cut-off of 75% of sucrose intake compared to baseline, we divided the animals into two groups: FS-vulnerable (FS-V), in which sucrose intake decreased by at least 25% from baseline, and FS-resilient (FS-R) (all the others) (Fig. 1C). Sucrose intake measured 24 h after FS remarkably decreased in FS-V rats compared to both control (CNT) and FS-R animals (Kruskal-Wallis *p* < 0.001; Dunn’s test: FS-V vs CNT *p* < 0.0001, FS-V vs FS-R *p* < 0.0001).

To assess whether different behavioral phenotypes were associated with changes in the stress response driven by the hypothalamic-pituitary-adrenal (HPA) axis at 24 h, we measured corticosterone serum levels (Fig. 1D), expression levels of mineralocorticoid (Fig. 1E) and glucocorticoid receptors (Fig. 1F), and phosphorylation of glucocorticoid receptors (Fig. 1G) in nuclear fractions of PFC from CNT, FS-R and FS-V rats sacrificed 24 h after stress. We found no significant changes among the three experimental groups (one-way ANOVA: MR F(2,26) = 0.4706, GR F(2,26) = 1.656, pGR F(2,26) = 1.679).

**Fig. 2:**
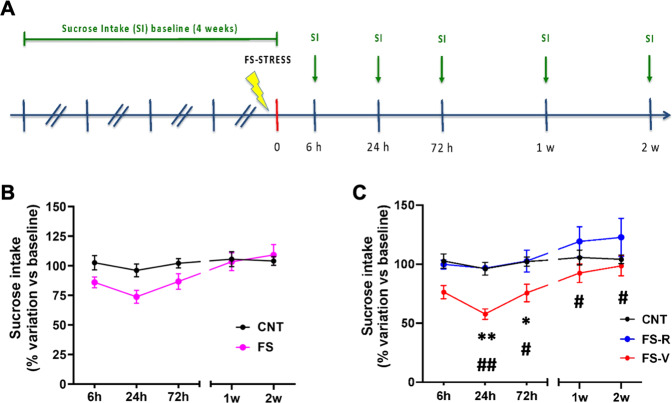
Time course of sucrose intake in FS-R and FS-V animals. **A** Experimental plan timeline. Basal sucrose intake was established as before, animals were subjected to a single session of FS, and sucrose intake test was performed 6 h, 24 h, 72 h, 1 week, and 2 weeks after stress exposure. **B** Time-course of sucrose intake in CNT and FS rats. Mixed-effects model, Šídák post-hoc test; *N* = 8 (CNT), 17 (FS). **C** Time-course of sucrose intake with separation in FS-R and FS-V. Mixed-effects model, Tukey’s post-hoc test: *p < 0.05, **p < 0.01, ^#^*p* < 0.05, ^##^*p* < 0.01; *N* = 8 (CNT), 7 (FS-R), 10 (FS-V).

We also investigated how long after stress exposure FS-R and FS-V animals maintained a difference in behavioral phenotype. We measured sucrose intake at different time-points after stress exposure in a dedicated set of animals (Fig. 2A). Taking all the stressed rats as a single group, mixed-effects model revealed only a significant effect of time (F(4,85) = 4.228), but not of stress (F(1,23) = 1.770) or time x stress interaction (F(4,85) = 1.643) (Fig. 2B). However, when FS-R and FS-V animals were segregated 24 h after stress as above, we observed that FS-V rats showed a trend for reduced sucrose intake compared to both CNT and FS-R animals already 6 h after stress while the reduction was significant 24 h and 72 h after FS (significant effect of time F(4,81) = 6.762 and stress F(2,22) = 10.54; Tukey’s test: 6 h: FS-V vs CNT *p* = 0.0592, FS-V vs FS-R *p* = 0.0727; 24 h: FS-V vs CNT *p* < 0.01, FS-V vs FS-R *p* < 0.01; 72 h: FS-V vs CNT *p* < 0.05, FS-V vs FS-R *p* < 0.05) (Fig. 2C). Moreover, FS-V animals still displayed a reduction of sucrose intake compared to FS-R animals 1 and 2 weeks after stress (Tukey’s test, 1 week: FS-V vs FS-R *p* < 0.05; 2 weeks: FS-V vs FS-R *p* < 0.05).

### Acute footshock stress induces selective changes of presynaptic glutamate release in the prefrontal cortex of FS-R and FS-V rats

**Fig. 3:**
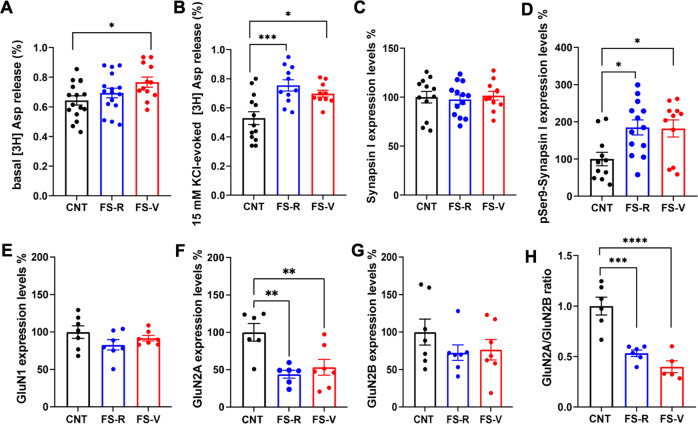
Glutamate release from PFC synaptosomes and protein expression and phosphorylation of AMPA and NMDA receptor subunits in PFC synaptosomes. **A** Basal [^3^H]-Asp release. One-way ANOVA, Tukey’s post-hoc test: *p < 0.05; *N* = 16 (CNT), 16 (FS-R), 12 (FS-V). **B** 15 mM KCl-evoked [^3^H]-Asp release. One-way ANOVA, Tukey’s post-hoc test: *p < 0.05, ***p < 0.001; *N* = 13 (CNT), 11 (FS-R), 10 (FS-V). **C** Total synapsin-I protein expression levels in PFC synaptic membranes. One-way ANOVA; *N* = 12 (CNT), 13 (FS-R), 11 (FS-V). **D** Phospho-Ser^9^ synapsin-I protein expression levels in PFC synaptic membranes. One-way ANOVA, Tukey’s post-hoc test: *p < 0.05; *N* = 11 (CNT), 13 (FS-R), 11 (FS-V). **E** GluN1, (**F**) GluN2A (**G**) GluN2B protein expression levels, and (**H**) GluN2A/2B ratio in PFC synaptic membranes. **E**, **H** One-way ANOVA, Tukey’s post-hoc test: ***p < 0.001, ****p < 0.0001. **F**, **G** Kruskal-Wallis, Dunn’s post-hoc test: **p < 0.01, *N* = 6/7 (CNT), 6/7 (FS-R), 6/7 (FS-V).

We have previously demonstrated that acute FS induces a rapid enhancement of depolarization-dependent (but not basal) presynaptic glutamate release in the PFC of rats, which lasts for up to 24 h after stress [10, 12, 13, 15]. Here we asked whether resilience or vulnerability to the effects of acute stress is associated with alterations of glutamate release 24 h after stress. We selected this time point for further experiments for two main reasons: (1) we were interested in early determinants of acute stress resilience/vulnerability; (2) 24 h was the timepoint when FS-V rats were in the larger number (58% of stressed rats). The spontaneous glutamate release, here referred as basal release, selectively increased in the PFC of FS-V rats compared to controls (one-way ANOVA F(2,41) = 3.379 *p* < 0.05; Tukey’s test: FS-V vs. CNT *p* < 0.05) (Fig. 3A). Instead, 15 mM KCl depolarization-evoked glutamate release increased similarly in both FS-R and FS-V (one-way ANOVA F(2,31) = 9.882 *p* < 0.001; Tukey’s test: FS-R vs CNT *p* < 0.001, FS-V vs CNT *p* < 0.05) (Fig. 3B).

The measurement of synapsin I expression and phosphorylation at Ser^9^ in PFC synaptic membranes confirmed our previous evidence showing that Ser^9^ phosphorylation is a key mechanism for increasing depolarization-evoked glutamate release after acute FS exposure [12, 13]. Indeed, while total synapsin I expression was unchanged (one-way ANOVA F(2,33) = 0.1543 *p* > 0.05) (Fig. 3C), the levels of phospho-Ser^9^-synapsin I increased in both FS-R and FS-V at 24 h (one-way ANOVA F(2,32) = 5.354 *p* < 0.01; Tukey’s test: FS-R vs CNT *p* < 0.05, FS-V vs. CNT *p* < 0.05) (Fig. 3D).

### Acute footshock stress induces changes in the expression of synaptic AMPA and NMDA receptors in the prefrontal cortex of FS-R and FS-V rats

To evaluate whether changes in presynaptic glutamate release were accompanied by postsynaptic alterations in AMPA and NMDA receptor levels and regulation, we measured protein expression and phosphorylation of main α-Ammino-3-idrossi-5-Metil-4-isossazol-Propionic Acid (AMPA) and N-Methyl-D-Aspartate (NMDA) receptor subunits in PFC from CNT, FS-R and FS-V animals 24 h after stress. We observed no differences in the total homogenate (Table 1). In synaptic membranes, we found no changes in GluA1 expression, phosphorylation at Ser^845^ and Ser^831^, GluA2 expression (Kruskal-Wallis *p* < 0.05 but no significant post-hoc tests) and phosphorylation at Ser^880^ (Table 2). On the other hand, while the expression levels of GluN1 (Fig. 3E) and GluN2B (Fig. 3G) were also unchanged (GluN1: one-way ANOVA F(2,18) = 1.695, *p* > 0.05; GluN2B: Kruskal-Wallis *p* > 0.05), GluN2A expression (Fig. 3F) and GluN2A/GluN2B ratio (Fig. 3H) were remarkably reduced in both FS-R and FS-V rats (GluNA2: one-way ANOVA F(2,16) = 9.008, *p* < 0.01; Tukey’s test: FS-R vs CNT *p* < 0.01, FS-V vs CNT *p* < 0.01; GluNA2/2B ratio: one-way ANOVA F(2,14) = 23.94, *p* < 0.0001; Tukey’s test: FS-R vs CNT *p* < 0.001, FS-V vs CNT *p* < 0.0001).

**Table 1:** Protein expression and phosphorylation of AMPA and NMDA receptor subunits in PFC homogenates.

	CNT	FS-R	FS-V	Statistics
GluN1	100 ± 12.49	63.86 ± 10.32	71.78 ± 11.09	ANOVA, F_(2, 23)_ = 2.77, *p* = 0.084
GluN2A	100 ± 11.65	66.09 ± 4.12	92.14 ± 13.95	Kruskal-Wallis, *p* = 0.128
GluN2B	100 ± 15.87	80.16 ± 8.81	87.80 ± 13.75	ANOVA, F_(2, 21)_ = 0.59, *p* = 0.564
GluN2A/GluN2B	100 ± 9.46	77.68 ± 5.42	84.68 ± 8.80	ANOVA, F_(2, 22)_ = 1.98, *p* = 0.162
GluA1	100 ± 12.34	75.64 ± 2.94	88.98 ± 5.65	ANOVA, F_(2, 22)_ = 2.09, *p* = 0.148
pSer^831^-GluA1	100 ± 10.08	99.90 ± 14.30	92.01 ± 6.59	ANOVA, F_(2, 22)_ = 0.18, *p* = 0.838
pSer^845^-GluA1	100 ± 16.22	86.94 ± 6.93	70.42 ± 7.73	Kruskal-Wallis, *p* = 0.262
GluA2	100 ± 7.33	86.81 ± 9.56	110.2 ± 9.58	ANOVA, F_(2, 24)_ = 1.69, *p* = 0.206
pSer^880^-GluA2	100 ± 10.84	91.16 ± 5.85	97.31 ± 7.32	ANOVA, F_(2, 24)_ = 0.55, *p* = 0.581

Data are reported as percentage compared to control (expression level of control sample is equal to 100) and as means ± SEM (*N* = 8–10). One-Way ANOVA or Kruskal-Wallis test.

**Table 2:** Protein expression and phosphorylation of AMPA receptor subunits in PFC synaptic membranes.

	CNT	FS-R	FS-V	Statistics
GluA1	100 ± 16.78	101.7 ± 18.75	75.25 ± 16.61	ANOVA, F_(2, 18)_ = 0.72, *p* = 0.498
pSer^831^-GluA1	100 ± 9.38	114.6 ± 20.29	90.78 ± 14.07	ANOVA, F_(2, 17)_ = 0.61, *p* = 0.556
pSer^845^-GluA1	100 ± 12.01	108.6 ± 18.70	92.98 ± 18.40	ANOVA, F_(2, 17)_ = 0.22, *p* = 0.806
GluA2	100 ± 16.06	54.79 ± 7.29	93.43 ± 10.27	Kruskal-Wallis, *p* = 0.042
pSer^880^-GluA2	100 ± 13.67	98.47 ± 17.97	72.79 ± 15.75	ANOVA, F_(2, 17)_ = 0.87, *p* = 0.438

Data are reported as percentage compared to control (expression level of control sample is equal to 100) and as means ± SEM (*N* = 8–10). Statistics: One-Way ANOVA or Kruskal-Wallis test.

**Fig. 4:**
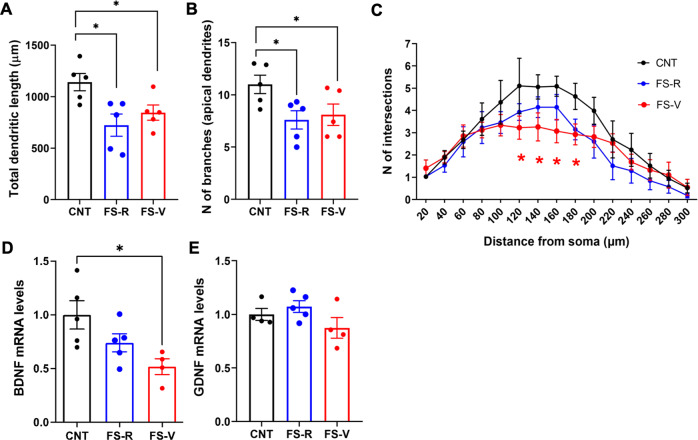
Dendritic arborization and neurotrophin expression in PFC. **A** Total length of PFC layers II–III pyramidal neurons apical dendrites. One-way ANOVA, Holm- Šídák post-hoc test: *p < 0.05. **B** Number of branches. One-way ANOVA, Holm- Šídák post-hoc test: *p < 0.05. **C** Sholl analysis. Mixed-effects model, Dunnett’s post-hoc test: *p < 0.05; *N* = 5 (CNT), 5 (FS-R), 5 (FS-V), 3–6 pyramidal neurons/animal. **D** BDNF and (**E**) GDNF mRNA expression levels in PFC. Kruskal-Wallis test, Dunn’s post-hoc test: *p < 0.05; *N* = 5 (CNT), 5 (FS-R), 5 (FS-V).

### Acute footshock stress induces changes in layers II/III dendrite morphology and neurotrophin levels in the prefrontal cortex of FS-R and FS-V rats

We have previously shown that acute FS induces a rapid retraction and simplification of prelimbic PFC layers II–III pyramidal neurons apical dendrites, which was measurable already after 24 h and sustained up to 14 days after stress [8, 15]. Here we found the total apical dendrite length (Fig. 4A) and number of dendritic branches (Fig. 4B) decreased in PFC layers II–III of both FS-R and FS-V rats (dendritic length: one-way ANOVA F(2,12) = 5.759, *p* < 0.05; Holm-Šídák test: FS-R vs CNT *p* < 0.05, FS-V vs CNT *p* < 0.05; N of branches: one-way ANOVA F(2,14) = 3.900, *p* < 0.05; Holm-Šídák test: FS-R vs CNT *p* < 0.05, FS-V vs CNT *p* < 0.05). However, Sholl analysis revealed a significant reduction in the number of intersections between 120 and 180 μm from soma only in FS-V animals compared to CNT (mixed-effects model, significant effect of distance F(14,189) = 20.58; Dunnett’s test: 120, 140, 160, 180 µm FS-V vs CNT *p* < 0.05) (Fig. 4C).

Local expression of neurotrophins has been linked to changes in dendrite development related to environmental factors, including exposure to stress [46–48]. Therefore, we measured the mRNA expression of the two main neurotrophins, Brain-Derived Neurotrophic Factor (BDNF) and Glial-Derived Neurotrophic Factor (GDNF). We found a significant reduction of total BDNF mRNA levels in the PFC of FS-V rats compared to CNT (Kruskal-Wallis *p* < 0.05; Dunn’s test: FS-V vs CNT *p* < 0.05) (Fig. 4D) and no changes of GDNF mRNA levels (Kruskal-Wallis *p* > 0.05) (Fig. 4E).

### Acute footshock stress induces changes in glutamate release from glial perisynaptic processes in the prefrontal cortex of FS-R and FS-V rats

An increasing number of studies suggest that synapses require astrocyte signals for optimal performances [26]. Indeed, gliotransmitters released by astrocytes modulate presynaptic efficacy and postsynaptic responses [49, 50]. Although this neuron-astrocyte crosstalk mainly bases on glutamate signaling [51], possible changes in glutamate release from perisynaptic astrocytic processes after FS were never assessed before.

**Fig. 5:**
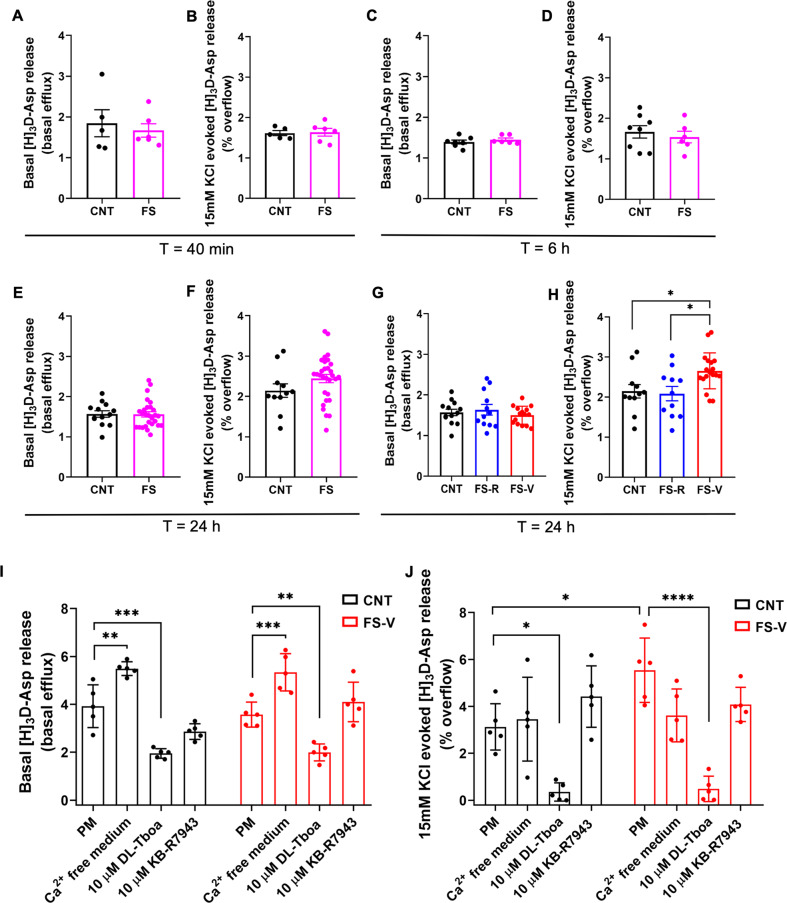
Glutamate release from PFC gliosomes. **A** Basal and (**B**) 15 mM KCl-evoked [^3^H]-Asp release measured immediately after FS (40 min from the beginning of stress). Unpaired student’s *t*-test; *N* = 5 (CNT), 6 (FS). **C** Basal and (**D**) 15 mM KCl-evoked [^3^H]-Asp release measured 6 h after the beginning of FS. Unpaired student’s *t*-test; *N* = 8 (CNT), 6 (FS). **E** Basal and (**F**) 15 mM KCl-evoked [^3^H]-Asp release measured 24 h after the beginning of FS. Unpaired student’s *t*-test; *N* = 11 (CNT), 30 (FS). **G** Basal and (**H**) 15 mM KCl-evoked [^3^H]-Asp release measured 24 h after the beginning of FS with separation in FS-R and FS-V. One-way ANOVA, Tukey’s post-hoc test: *p* < 0.05. *N* = 11 (CNT), 11 (FS-R), 19 (FS-V), *p < 0.05. **I** Basal and (**J**) 15 mM KCl-evoked [^3^H]-Asp release measured in physiological conditions (physiological medium, PM) or in presence of calcium free medium, 10 µM DL-TBOA or 10 µM KB-R7943 24 h after the beginning of FS in CNT and FS-V. Two-way ANOVA, Tukey’s post-hoc test: *p* < 0.05. *N* = 5 (CNT), 5 (FS-V), *p < 0.05, **p < 0.01, ***p < 0.001 and ****p < 0.0001.

Here, for the first time we measured glutamate release from PFC glial perisynaptic processes (gliosomes) at different time points after acute FS. We did not find any difference in basal and 15 mM KCl depolarization-evoked glutamate release between control and FS animals immediately after stress (Student’s *t*-test; basal release: *t* = 0.5060, *p* > 0.05; depolarization-evoked release: *t* = 0.1749, *p* > 0.05) (Fig. 5A, B), as well as 6 h after stress (Student’s *t*-test; basal release: *t* = 0.9130, *p* > 0.05; depolarization evoked release: *t* = 0.5830, *p* > 0.05) (Fig. 5C, D) or 24 h after the start of stress (Student’s *t*-test; basal release: *t* = 0.07739, *p* > 0.05; depolarization evoked glutamate release: *t* = 1.501, *p* > 0.05) (Fig. 5E, F). However, when we separated rats into FS-R and FS-V, 24 h after stress, basal glutamate release from PFC gliosomes was unchanged (one-way ANOVA, F(2,37) = 0.5652, *p* > 0.05) (Fig. 5G) while depolarization-evoked glutamate release was significantly increased only in FS-V compared to both FS-R and control rats (one-way ANOVA, F(2,38) = 5.519, *p* < 0.05; Tukey’s test: FS-V vs CNT *p* < 0.05, FS-V vs FS-R *p* < 0.05) (Fig. 5H).

To understand the mechanisms underlying the increased glutamate release in PFC gliosomes of FS-V animals, we tested the involvement of calcium (Ca^2+^) and glutamate transporters. We found the basal glutamate release significantly increased in the absence of Ca^2+^. Still, it was strongly reduced by 10 µM DL-Tboa, a blocker of glutamate transporters [52], and unchanged in the presence of 10 µM KB-R7943, a blocker of the Na^+^/Ca^2+^ exchangers [53], when working in a reverse mode, in both control and FS-V rats (two-way ANOVA, significant effect of treatment F(3,32) = 58.72, *p* < 0.0001 and of stress x treatment interaction F(3,32) = 3.750, *p* < 0.05; Tukey’s test: CNT: Ca^2+^-free vs physiological medium (PM) *p* < 0.01, DL-Tboa vs. PM *p* < 0.001; FS-V: Ca^2+^-free vs PM *p* < 0.001, DL-Tboa vs PM *p* < 0.01) (Fig. 5I). These experiments showed that spontaneous glutamate release from PFC gliosomes is mediated by glutamate transporters.

Notably, 10 µM DL-Tboa also abolished the depolarization-evoked glutamate release in both control and FS-V animals (in which glutamate release was confirmed to be higher compared to controls), suggesting a complete dependence on glutamate transporters (two-way ANOVA, significant effect of treatment F(3,32) = 27.22, *p* < 0.0001 and of stress x treatment interaction F(3,32) = 3.041, *p* < 0.05; Tukey’s test: FS-V vs PM *p* < 0.05; CNT: DL-Tboa vs PM *p* < 0.05; FS-V: DL-Tboa vs PM *p* < 0.0001). At the same time, no effects of Ca^2+^-free medium or KB-R7943 were measured (Fig. 5J).

### Acute footshock stress induces changes in astrocyte proteins related to glutamate homeostasis in the prefrontal cortex of FS-R and FS-V rats

**Fig. 6:**
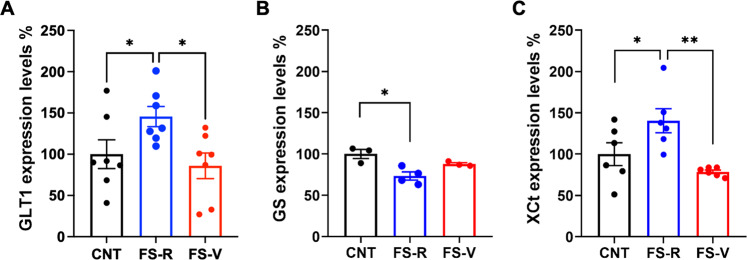
Expression of astrocytic proteins related to glutamate homeostasis in PFC gliosomes. **A** Glutamate transporter-1 (GLT1), (**B**) glutamine synthetase (GS), and (**C**) cystine/glutamate antiporter (xCt) protein expression in PFC gliosomes. **A** one-way ANOVA, Holm-Šídák test: *p < 0.05; *N* = 7 (CNT), 7 (FS-R), 3 (FS-V). **B** Kruskal-Wallis test, Dunn’s post-hoc test: *p < 0.05; *N* = 3 (CNT), 4 (FS-R), 3 (FS-V). **C** one-way ANOVA, Holm-Šídák test: *p < 0.05, **p < 0.01; *N* = 6 (CNT), 6 (FS-R), 6 (FS-V).

Protein expression levels of glutamate transporter 1 (GLT1), glutamine synthetase (GS), and cysteine-glutamate exchanger (xCt) were unchanged in PFC total homogenates (Table 3). In gliosomes from FS-R rats, GLT1 expression was increased compared to both CNT and FS-V rats (one-way ANOVA F(2,18) = 4.181 *p* < 0.05; Holm-Šídák test: FS-R vs CNT *p* < 0.05, FS-V vs FS-R *p* < 0.05) (Fig. 6A) and GS was reduced compared to CNT (Kruskal-Wallis *p* < 0.01; Dunn’s test: FS-R vs CNT *p* < 0.05) (Fig. 6B). xCt was increased compared to both CNT and FS-V animals (one-way ANOVA F(2,15) = 7.216 *p* < 0.01; Holm-Šídák test: FS-R vs CNT *p* < 0.05, FS-V vs FS-R *p* < 0.01) (Fig. 6C).

**Table 3:** Expression of astrocytic proteins related to glutamate homeostasis in PFC homogenates.

	CNT	FS-R	FS-V	Statistics
GLT1	100 ± 7.84	105.79 ± 12.14	84.38 ± 4.68	ANOVA, F_(2, 13)_ = 1.536, *p* = 0.251
GS	100 ± 3.52	117.04 ± 9.34	98.28 ± 5.10	ANOVA, F_(2, 13)_ = 2.621, *p* = 0.110
xCt	100 ± 10.91	110.24 ± 6.52	113.52 ± 6.46	Kruskal-Wallis, *p* = 0.396

Data are reported as percentage compared to control (expression level of control sample is equal to 100) and as means ± SEM (*N* = 4–6). One way ANOVA or Kruskal-Wallis test.

## Discussion

In the present study, we report the first results obtained with a new rodent model of acute stress, envisaged to identify early determinants of resilient vs. vulnerable trajectories of the stress response [2, 20]. The model is based on a standard protocol of acute FS stress, which has previously allowed us a thorough dissection of both early and long-term consequences of acute inescapable stress exposure [8, 10, 12–16]. Here, for the first time, by measuring structural, functional, and molecular readouts 24 h after stress exposure, we identified changes that we interpreted as early determinants of resilient vs. vulnerable stress responses.

The results can be summarized as follows:

- 24 h after exposure to acute FS, rats can be deemed FS-R or FS-V based on their anhedonic phenotype in the sucrose intake test;
- FS-R and FS-V rats are phenotypically distinguishable up to two weeks after stress exposure;
- basal neuronal glutamate release increased in the PFC of FS-V rats, while depolarization-evoked glutamate release and synapsin I phosphorylation at Ser^9^ increased in both FS-R and FS-V animals;
- FS decreased the synaptic expression levels of the GluN2A subunit in the PFC and reduced apical dendritic length and complexity of prelimbic PFC layers II–III pyramidal neurons (both FS-R and FS-V);
- BDNF expression selectively diminished in the PFC of FS-V rats;
- Depolarization-evoked (carrier-mediated) glutamate release from astroglia perisynaptic processes (gliosomes) selectively augmented in the PFC of FS-V rats;
- GLT1 and xCt levels increased while GS expression diminished in purified gliosomes from the PFC of FS-R rats.

Table 4 summarizes the main functional and molecular differences highlighted between FS-R and FS-V animals.

**Table 4:** Summary of functional, molecular, and structural differences between FS-R and FS-V animals in PFC synaptosomes, gliosomes, and whole tissue.

PFC SYNAPTOSOMES	FS-R	FS-V
**Basal glutamate release**	**=**	**↑**
Depolarization-evoked glutamate release	**↑**	**↑**
pSer^9^-synapsin I protein levels (synaptic membranes)	**↑**	**↑**
GluN2A protein levels and GluN2A/GluN2B ratio (synaptic membranes)	**↓**	**↓**

PFC GLIOSOMES		
Basal glutamate release	**=**	**=**
**Depolarization-evoked glutamate release**	**=**	**↑**
**GLT1 protein levels**	**↑**	**=**
**GS protein levels**	**↓**	**=**
**XCt protein levels**	**↑**	**=**

TOTAL PFC		
Dendritic length and branching (prelimbic PFC layers II–III pyramidal neurons)	**↓**	**↓**
**BDNF mRNA levels**	**=**	**↓**
GDNF mRNA levels	**=**	**=**

Arrows/equals represent the comparison vs control animals.

The parameters which were found different between FS-R and FS-V rats are highlighted in bold.

### Sucrose intake test allows deeming rats resilient or vulnerable to acute footshock stress

Anhedonia, the disruption of anticipation, motivation, and decision-making processes involved in obtaining a reward, is a core symptom of depression and other neuropsychiatric disorders [54, 55]. Typically, the sucrose intake test is used to assess the development of anhedonic phenotype induced by chronic stress [56, 57]. Here, we set up a novel paradigm based on differences in sucrose intake in acutely stressed rats, revealing the development of an anhedonic phenotype in about half of the stressed rats. We found no significant changes at the level of HPA axis activation 24 h after stress exposure, in line with previous evidence [1].

We demonstrated that FS-V animals remained anhedonic for 72 h after stress while, interestingly, FS-R and FS-V animals showed different behavioral phenotypes at least up to 2 weeks after acute FS exposure. These duration differences suggest that this paradigm generates different trajectories of resilient versus vulnerable stress response, thus allowing the dissection of the response and its readouts [2, 20]. Future studies are needed to draw a comprehensive time-dependent behavioral characterization of FS-R and FS-V animals in the days and weeks following stress exposure, thus analyzing specific behavioral phenotypes which could clarify the translational relevance of the model.

### Advantages in studying functional changes at glutamatergic tripartite synapses using purified synaptosomes and gliosomes in superfusion

The anatomic and functional assembly of astrocytes with the presynaptic and post-synaptic neuronal domains led to the widely accepted concept of the tripartite synapse [25, 26, 28]. As for functional characterization, we focused on glutamatergic synapses and dissected neuronal and glial determinants in response to acute stress by studying glutamate release from synaptosomes and gliosomes. Synaptosomes derive from synaptic boutons and varicosities of the axonal processes, maintain the complexity of the presynaptic nerve terminals, and represent the site of neurotransmitter release [58–60]. Gliosomes are a subcellular preparation originating from the perisynaptic astrocytes’ regions [35, 61]. They express proteins involved in the release machinery, and contain vesicles competent for exocytosis, neurotransmitter receptors, and transporters [62, 63].

Synaptosomes and gliosomes have several advantages: (i) they can be rapidly and easily prepared; (ii) they originate from mature neurons and astrocytes; (iii) they retain the functional modifications induced in vivo by environmental or pharmacological stimulation.

Moreover, we measured glutamate release under superfusion conditions, a technique avoiding cellular consequences up- or downstream neurotransmitter release [27, 41]. During superfusion, released transmitters are immediately removed before they are taken up by transporters or interact with receptors expressed at the presynaptic level. Consequently, superfusion conditions allow us to measure the actual glutamate release, not influenced by reuptake processes. During superfusion, indirect effects due to released compounds from adjacent structures also appear minimized. Therefore, the modification of glutamate release observed after exposure to stress can be ascribed to plastic changes induced by stress selectively at glutamate releasing synaptosomes or gliosomes [64–66].

### Neuronal glutamate homeostasis is altered in the prefrontal cortex of rats vulnerable to acute stress

We previously characterized the PFC time-dependent functional alterations induced by acute FS stress. While revealing a rapid and sustained increase of depolarization-evoked glutamate release up to 24 h after stress with a mechanism dependent on synapsin I Ser^9^ phosphorylation, as confirmed here, we reported no effects on the spontaneous glutamate release [8, 10, 12–16]. Conversely, the present study, deeming rats FS-R or FS-V, highlighted a selective increase of basal neuronal glutamate release in FS-V animals.

Basal synaptosomal glutamate release is a measure of resting glutamate release in-situ, dependent on spontaneous synaptic vesicle fusion at synapses [67, 68]. The spontaneous release of glutamate plays a central role in the maturation and stability of synaptic networks, controlling spike timing, maintaining synaptic strength, and regulating postsynaptic responsiveness [69–71]. Interestingly, the spontaneous release has been proposed to inhibit dendritic protein translation [72, 73]. Although further studies are needed to understand the functional consequences of increased basal glutamate release in the PFC of FS-V rats, our findings suggest that in these animals the mechanisms that regulate glutamate homeostasis fail to properly restore glutamatergic activity after exposure to stress, with possible impact on the level of dendritic protein synthesis.

On the other hand, the reduction of GluN2A expression and GluN2A/B ratio in PFC synaptic membranes of stressed rats is suggestive of an adaptation to excessive stimulation, with a relative increase of extrasynaptic GluN2B-containing NMDA receptors and reduced postsynaptic activity [74]. Although electrophysiological measurements are needed to confirm these data, this finding seems in line with a switch between synaptic and extrasynaptic NMDA receptors after stress [75].

### Dendritic simplification and reduced BDNF expression in the prefrontal cortex may contribute to acute stress vulnerability

We confirmed here that acute FS stress induces a shortening and simplification of apical dendrites in prelimbic PFC layers II–III pyramidal neurons as early as 24 h after stress exposure [8]. Although total dendritic length and number of branches were similar in FS-R and FS-V rats, Sholl analysis highlighted a significant reduction in the number of intersections between 120 and 180 μm from soma exclusively in FS-V animals, suggesting subtle differences in dendritic remodeling between the two groups.

Observing dendritic retraction and simplification in FS-R rats is not surprising. Indeed, neuroarchitecture changes in response to stress are a physiological mechanism in the adult brain required for adaptation and learning [1, 20]. Accordingly, we could hypothesize that dendritic retraction early after stress represents an adaptive mechanism to protect neurons from glutamate release induced by stress, while in the long-term, resilience implies the recovery from stress-induced changes in neuronal architecture [1, 2]. It will be interesting to study morphological alterations in the two phenotypes at additional time points after FS stress exposure to confirm this hypothesis since we have previously shown that dendritic retraction induced by acute stress lasts up to 14 days [8].

Moreover, we measured a significant reduction of BDNF mRNA expression in the PFC of FS-V rats but not in FS-R. BDNF plays a crucial role in the activity-dependent regulation of synaptic homeostasis and its expression has been consistently reported to be reduced in corticolimbic areas after chronic stress, while proadaptive treatments rescue this reduction [2, 76]. Recent evidence suggests that BDNF plays a role in fear memory consolidation and extinction learning, orchestrating sensitivity to stress, trauma, and risk of stress-related psychiatric disorders [77]. Furthermore, BDNF/trkB signaling has been critically involved in stress-induced depressive-like behavioral abnormalities, including anhedonic behavior [78]. Thus, a reduction of BDNF expression in acute stress vulnerable animals may participate in maladaptive stress response mechanisms. However, further studies are required to understand the possible functional consequences of reduced BDNF mRNA levels measured in FS-V and to test the effects of treatment strategies able to normalize BDNF levels. In this context, it would be interesting to consider not only traditional antidepressants, but also acute subanesthetic ketamine, which we have shown can restore long-term glutamatergic alterations and dendritic atrophy induced by FS in PFC [10, 14, 15].

### Perisynaptic astrocyte dysfunction is involved in the maladaptive response to acute stress

In the present study, we found that, differently from synaptosomes, basal gliosome glutamate release was unchanged, while depolarization-evoked glutamate release was significantly increased in FS-V (but not in FS-R rats) exclusively 24 h after stress. Overall, our release experiments revealed that the activity-dependent glutamate release from presynaptic nerve terminals in the PFC immediately and persistently increased after acute stress exposure. In contrast, the perisynaptic astrocytic depolarization-evoked glutamate release increased in FS-V rats, and it was temporally delayed and perhaps subordinate to the neuronal counterpart.

Looking at the mechanisms of glutamate release from gliosomes, we observed that the absence of external Ca^2+^significantly increased basal release in both FS-R and FS-V rats. We hypothesize that this condition can alter the transmembrane gradient, forcing Na^+^/Ca^2+^ exchangers to extrude Ca^2+^ in exchange for Na^+^, favoring membrane depolarization and glutamate release. On the other hand, the depolarization-evoked glutamate release was entirely dependent on glutamate transporters. Glutamate release from astrocytes may occur either from vesicular stores or by transporter reversal [79]. In our case, the reversal of transporters can be provoked by the increase of cytoplasmic Na^+^ concentration and the decrease of Na^+^-gradient [80–82]. Blocking glutamate transporters also partly but significantly reduced basal gliosome glutamate release, suggesting a direct role of the metabolic pool of cytoplasmic glutamate [79].

We also obtained evidence of expression changes of astrocyte proteins related to glutamate homeostasis. In gliosomes of FS-R animals, the expression of GLT1, the principal astrocyte transporter responsible for glutamate removal [83], was significantly increased. This increase is in line with previous evidence showing an increase in GLT1 upon acute stress exposure [84, 85]. On the other hand, the lack of GLT1 increase in FS-V rats is suggestive of lack of efficiency in facing stress insults. Moreover, the finding that depolarization-evoked glutamate release increases in gliosomes from FS-V rats by the reversal of glutamate transporters working in the in-out direction may suggest changes in the function of quantitatively unmodified GLT1 transporters.

The expression of GS, the astrocyte glutamate-degrading enzyme [83], decreased selectively in gliosomes from FS-R rats. This result was unexpected because the increase or maintenance of GS function has been shown to promote protective effects against excessive glutamate release [86].

Also, the expression of xCT, an antiporter that astrocytes use to extrude glutamate [83], was increased in gliosomes of FS-R but not in gliosomes of FS-V rats. In line with the present results, increased xCT activity, contributing to priming glutamate homeostasis, is implicated in mechanisms of stress resilience and antidepressant-like response [87].

## Conclusion

In the present study, we show for the first time that the application of the sucrose intake test to rats exposed to acute inescapable FS stress allows the generation of a novel animal model of resilience/vulnerability to acute stress. The purification of synaptosomes and gliosomes allowed for studying functional and molecular changes of the synaptic and perisynaptic moieties of the tripartite glutamatergic synapse in the PFC of vulnerable and resilient animals. Our structural, functional, and molecular studies revealed an excess of the basal presynaptic and depolarization-dependent perisynaptic glutamate release in the PFC of FS-V rats, supported by changes in the expression of BDNF and astrocyte homeostasis-related proteins, contributing to the glutamatergic dysfunction in these animals. The PFC has been selected here essentially because in our previous studies we found rapid functional, structural, and molecular changes in this brain area, which is known to play key roles in executive function and extinction of fear learning [8, 10–14, 16]. Nevertheless, we can’t exclude that other brain areas could contribute to the development of resilient or vulnerable phenotypes.

Noteworthy, this study has been conducted on male rats only, even though it is known that women are more susceptible to stress-related adverse outcomes [88–91]. Sex differences may be expected in the adaptive/maladaptive response to acute traumatic stress and recent evidence highlighted sex differences in fear extinction in rodents [92–94]. More studies are required to investigate sex-specific pathophysiological mechanisms in the stress response.

Despite these limitations, in the present study, we found that PFC synaptosomes and gliosomes from male rats vulnerable and resilient to acute FS displayed a different response to stress, suggesting that neurons undergo early modifications after FS, with astroglia entering the play later, thus suggesting that the observed astroglia modification may be primed by neuronal modification. Alternatively, the molecular changes occurring in PFC gliosomes might be responsible for adaptation, thus assigning astrocytes a central role in avoiding a maladaptive response progression in FS-R rats. To detect why this astrocytic attempt failed in FS-V rats could reveal new mechanisms involved in PTSD and deserves further investigation. Overall, we could identify early determinants of a maladaptive response leading to behavioral vulnerability to stress. The study of these mechanisms promises to help in the identification of key mediators of pro-adaptive versus maladaptive trajectories.

## Supplementary information

Supplementary Materials and Methods
