# Supplementary material for: Changes at glutamate tripartite synapses in the prefrontal cortex of a new animal model of resilience/vulnerability to acute stress
Source: Transl Psychiatry. 2023 Feb 18;13:62. doi: 10.1038/s41398-023-02366-w (PMC9938874; doi:10.1038/s41398-023-02366-w)
Supplement: Supplementary file 1 — Supplementary Materials and Methods [file 41398_2023_2366_MOESM1_ESM.docx]

**Supplementary Information**

**Supplementary Materials and methods**

**Animals**

All experimental procedures involving animals were performed following the European Community Council Directive 2010/63/UE and were approved by the Italian legislation on animal experimentation (Decreto Legislativo 26/2014, animal experimentation licenses N 521/2015-PR and 140/2014-B—DGSAF24898). Experiments were performed with male Sprague-Dawley rats (175–200 g in weight at the beginning of the protocol, 350–450 g at the end). All the animals were housed two per cage (except during the sessions of sucrose intake test) and maintained on a 12/12 h light/dark schedule (lights on at 7:00 am), in a temperature- and humidity-controlled facility with free access to food and water. All the animals were sacrificed by beheading.

**Sucrose intake test and classification of resilient and vulnerable animals**

Sucrose intake was evaluated as in [1]. After 1 week of acclimatization, rats were habituated to a palatable sweet solution by removing water and exposing them to two bottles with 1% sucrose solution for 2 h. Starting from the day after habituation, animals were exposed to sucrose twice a week for 4 weeks, according to the following protocol: rats were single housed, provided with two bottles, one containing 1% sucrose and one containing tap water (the position of the bottles was inverted after 30 min), with no food pellet, for 1 h. Animals were not food and/or water deprived before the test. The average sucrose solution volume drunk by each animal was calculated and defined as baseline sucrose intake. After 4 weeks, animals were randomly assigned to FS (see below) or left undisturbed in their home cages (control). Sucrose intake test was repeated the day after (sucrose intake test started 23 h after the beginning of FS) and percent sucrose intake vs. baseline sucrose intake for each animal was calculated. Animals showing at least a 25% within-subject decrease in sucrose intake were considered anhedonic and classified as vulnerable (FS-V), while all the others were defined as resilient (FS-R). The animals were sacrificed immediately after the sucrose intake test except for one subset, in which we analyzed the time course of the anhedonic phenotype, by measuring sucrose intake 6 h, 24 h, 72 h, 1 week and 2 weeks after FS. Experimenters were not blinded to the group allocation.

**Footshock stress procedure**

Animals were subjected to a single session of acute inescapable FS stress as previously reported [1]: intermittent shocks (0.8 mA) for 40 min (20 min total of actual shock with random intershock length between 2 and 8 s). The FS box was connected to a scrambler controller (LE 100-26, Panlab) that delivers intermittent shocks to the metallic floor. Control animals were left undisturbed in their home cages.

**Serum corticosterone assay**

Trunk blood was collected immediately after sacrifice and centrifuged at room temperature for 20 min at 3000g. The supernatant, separated from blood cells and coagulation factors, was aliquoted and stored at -80 °C. Blood Serum corticosterone levels were measured using a commercial kit (Corticosterone EIA kit, Enzo Life Sciences Inc., Farmingdale, NY) as previously reported [2].

**Preparation of subcellular fractions**

Immediately after sacrifice, the PFC has been rapidly collected in ice, homogenized in 10 volumes of Tris-buffered 0.32 M sucrose, and centrifuged at 1,000g for 5 min to obtain the nuclear fraction [3]. Purified synaptic terminals (synaptosomes) and glial perisynaptic processes (gliosomes) were purified on a discontinuous Percoll^®^ gradients in Tris-buffered 0.32 M sucrose as previously described [1,4,5]. Synaptic membranes were prepared as in [6].

When used for neurotransmitter release experiments, synaptosomes and gliosomes were resuspended in a physiological medium (PM) having the following composition (140 mM NaCl, 3 mM KCl, 1.2 mM MgSO_4_, 1.2m M CaCl_2_, 1.2 mM NaH_2_PO_4_, 5 mM NaHCO_3_, 10 mM glucose, 10 mM HEPES, pH 7.4). For western blotting experiments, the nuclear fraction, synaptosomes, synaptic membranes and gliosomes were resuspended in lysis buffer (120 mM NaCl, 20 mM HEPES, 0.1 mM EGTA, 0.1 mM DTT, phosphatase (Thermo Fisher Scientific, Milano, Italy) and protease (Sigma-Aldrich, Milano, Italy) inhibitors) [3,7,8].

**Measurement of neurotransmitter release from purified synaptosomes and gliosomes**

Synaptosomes and gliosomes were labeled with 0.05µM [^3^H]_D_-Aspartate (a non-metabolizable analogous of glutamate used to label glutamate releasing pools; [9]). Aliquots were distributed on microporous filters placed at the bottom of a set of 24 parallel superfusion chambers maintained at 37 °C (Superfusion System, Ugo Basile, Comerio, Varese, Italy) and processed as previously described [7,10]. Superfusion was started with physiological medium at a rate of 0.5 ml/min and continued for 48 min. After 39 min of superfusion to equilibrate the system, stimulation with a 90 s pulse of 15 mM KCl was applied, with KCl substituting for an equimolar concentration of NaCl. When appropriate, Ca^2+^ free medium was added to the superfusion medium at t = 20 min and maintained until the end of the experiments. 10 µM DL-Tboa (a competitive, non-transportable blocker of excitatory amino acid transporters) or 10 µM KB-R7943 (a blocker of Na^+^/Ca^2+^ exchanger working in the reverse mode) were added at t =30 min until the end of the experiments. Two 3 min samples (t = 36–39 and 45–48 min; basal release) and one 6 min sample (t = 39–45 min; stimulus-evoked release) were collected. At the end of superfusion, samples and superfused synaptosomes/gliosomes were counted for radioactivity. Tritium released in each sample collected was calculated as fractional rate x 100 (basal efflux; percentage of the total synaptosomal/gliosomal tritium content at the beginning of the respective collection period). The stimulus-evoked overflow was estimated by subtracting the transmitter content in the two 3 min fractions, representing the basal release, from the 6 min fraction collected during and after the stimulation pulse. Synaptosomes/gliosomes from the same animal were loaded on 4 independent chambers and release results were averaged.

**Western blotting**

Protein concentration was calculated by Bradford or BCA assays (Sigma-Aldrich, Milano, Italy and Thermo-Fisher Scientific, Milano, Italy, respectively) and 10-30 micrograms were loaded on acrylamide SDS-PAGE gels. Western blotting was performed as previously described [8,11]. Primary antibodies used were against mineralocorticoid receptor (MR) (1:1,000, Santa Cruz Biotechnology, Dallas, TX, cod. sc-11412), glucocorticoid receptor (GR) (1:1,000, Santa Cruz Biotechnology, cod. sc-393232), phospho-Ser^203^-GR (1:1,000, Cell Signaling Technology, Danvers, MA, cod. #4161), synapsin I (1:4,000, Synaptic Systems, Goettingen
Germany, cod. 106 001), phospho-Ser9-synapsin I (1:1,000, Cell Signaling Technology, cod. #2311), α-Ammino-3-idrossi-5-Metil-4-isossazol-Propionic Acid (AMPA) receptor subunit A1 (GluA1) (1:200, Alomone Labs, Jerusalem, Israel, cod. AGC-004), A2 (GluA2) (1:2,500, Alomone Labs, Cod. AGC-005), phospho-Ser^831^-GluA1 (1:1,000, Abcam, Cambridge, United Kingdom, cod. Ab109464), phospho-Ser^845^-GluA1 (1:1,000, Abcam, cod. ab3901), phospho-Ser^880^-GluA2 (1:1,000, Abcam, cod. Ab52180), N-Methyl-D-Aspartate (NMDA) receptor subunit 1 (GluN1) (1:500, Merck-Millipore, Milano, Italy, cod. AB9864), 2A (GluN2A) (1:500, Merck-Millipore, cod. AB1555P), 2B (GluN2B) (1:500, Merck-Millipore, cod. 454,582), glutamine synthetase (GS) (1:1,000, Merck-Millipore, cod. MAB302), glutamate transporter-1 (GLT1) (1:500, Santa Cruz Biotechnology, cod. sc-365634), cystine/glutamate antiporter (xCt) (1:1,000, Novus Biologicals, Littleton, CO, USA, cod. NB300-318), Glyceraldehyde-3-Phosphate Dehydrogenase (GAPDH) (1:8,000, Merck-Millipore, cod. Mab374), and β-Actin (1:40,000, Merck-Millipore, cod. A1978). Secondary antibodies used were anti-mouse and anti-rabbit horseradish peroxidase (HRP)-conjugated (1:10,000, Jackson ImmunoResearch, Suffolk, UK) or fluorophor-conjugated antibodies (1:2,000, IRDye 800CW goat anti-rabbit IgG or IRDye 680RD goat anti-mouse IgG, LI-COR). Signals were detected using an enhanced chemiluminescence (ECL) kit (GE Healthcare Life Sciences, Milan, Italy), visualized with Chemidoc XRS and Image Lab software (Bio-Rad Laboratories, Milano, Italy), and quantified with ImageJ [12], or detected with Odyssey infrared imaging system (LI-COR Biosciences, Lincoln, NE, USA) and quantified with Image Studio software (version 5.2, LI-COR Biosciences) [13]. Total protein content, GAPDH, or β-Actin expression levels were used for normalization. The levels of phosphorylated proteins were normalized to respective total protein levels [11]. Each sample was loaded twice and run in at least 2 independent experiments.

**Golgi-Cox staining and dendritic analysis**

Golgi-Cox staining was performed using the Rapid Golgi Stain Kit (FD NeuroTechnologies, Inc., Columbia, MD, United States) on a dedicated set of animals as previously reported [1]. Brains were coronally sliced (200 μm) on a cryostat (Leica CM1950, Leica Biosystems, Buccinasco, Italy). Pyramidal neurons within prelimbic PFC layers II–III were identified and, using a ×40 objective, Z-stacks (80–100 μm; Z-step size 1 μm) of 3–6 pyramidal neurons/animal with untruncated branches were acquired (Nikon ViCo, Nikon Instruments S.p.A., Firenze, Italy).

Collapsed Z-stacks were imported in the open-source Fiji software [14] and dendrites were reconstructed using “Simple Neurite Tracer” Fiji plugin [15]. Dendritic length and branching, and Sholl analysis were assessed. Three blinded observers processed all the images.

**Real time-quantitative polymerase chain reaction (RT-qPCR)**

RT-qPCR was performed as previously described [16,17]. Briefly, total RNA was extracted from PFC using TRI-Reagent (Sigma-Aldrich) and quantified by D30 BioPhotometer spectrophotometer (Eppendorf AG, Hamburg, Germany). QuantiTect Reverse Transcription Kit (Qiagen, Valencia, CA, USA) was used for retrotranscription. qPCR was performed using iTaq Universal SYBR Green supermix (Bio-Rad Laboratories), using the CFX96 Touch thermocycler (Bio-Rad Laboratories). The mean of hypoxanthine-guanine phosphoribosyltransferase (Hprt) and TATA-box binding protein (Tbp) was used for normalization. Data were analyzed using the Pfaffl method, considering the efficiencies of the primers used (Pflaffl, 2001). Primers sequences used were: Bdnf: forward primer (fwd) 5’- GGGACTCTGGAGAGCGTGAA - 3’; reverse primer (rev) 5’- GTCAGACCTCTCGAACCTGC - 3’; Gdnf: fwd 5’- CACCAGATAAACAAGCGGCG - 3’; rev 5’- TCGTAGCCCAAACCCAAGTC - 3’; Hprt: fwd 5’- TCCCAGCGTCGTGATTAGTGA - 3’; rev 5’- CCTTCATGACATCTCGAGCAAG - 3’; Tbp: fwd 5’- TGGGATTGTACCACAGCTCCA - 3’; rev 5’- CTCATGATGACTGCAGCAAACC - 3’. Samples were analyzed in triplicates.

**Statistical analysis**

Statistical data analysis was carried out using GraphPad Prism 9 (GraphPad Software Inc., USA). Results are presented as means ± standard error of the mean (SEM).

Normal distribution was verified using Kolmogorov–Smirnov test. For normally distributed data, statistical analyses were performed with unpaired Student's t-test, one or two-way analysis of variance (ANOVA), and mixed-effects model when appropriate, followed by post-hoc multiple comparison tests as indicated in the figure legends. For non-normally distributed data, statistically analyses were performed with the Mann Whitney test (when 2 groups were compared) or Kruskal-Wallis test followed by Dunn's multiple comparison test. F test was applied to compare variances within each group, p>0.05.

The number of animals used in each experiment is indicated in the figure legends and sample size was calculated based on previous experiments in order to have a power >80% to detect differences >30% at SD of 25% and alpha error of 5%.

**References**

1. Sala N, Paoli C, Bonifacino T, Mingardi J, Schiavon E, la Via L, et al. Acute Ketamine Facilitates Fear Memory Extinction in a Rat Model of PTSD Along With Restoring Glutamatergic Alterations and Dendritic Atrophy in the Prefrontal Cortex. Front Pharmacol. 2022;13:759626.

2. Musazzi L, Sala N, Tornese P, Gallivanone F, Belloli S, Conte A, et al. Acute Inescapable Stress Rapidly Increases Synaptic Energy Metabolism in Prefrontal Cortex and Alters Working Memory Performance. Cereb Cortex. 2019;29:4948–57.

3. Musazzi L, Seguini M, Mallei A, Treccani G, Pelizzari M, Tornese P, et al. Time-dependent activation of MAPK/Erk1/2 and Akt/GSK3 cascades: modulation by agomelatine. BMC Neurosci. 2014;15:119.

4. Stigliani S, Zappettini S, Raiteri L, Passalacqua M, Melloni E, Venturi C, et al. Glia re-sealed particles freshly prepared from adult rat brain are competent for  exocytotic release of glutamate. J Neurochem. 2006;96:656–68.

5. Ravera S, Torazza C, Bonifacino T, Provenzano F, Rebosio C, Milanese M, et al. Altered glucose catabolism in the presynaptic and perisynaptic compartments of SOD1 G93A mouse spinal cord and motor cortex indicates that mitochondria are the site of bioenergetic imbalance in ALS. J Neurochem. 2019;151:336–50.

6. Treccani G, Musazzi L, Perego C, Milanese M, Nava N, Bonifacino T, et al. Stress and corticosterone increase the readily releasable pool of glutamate vesicles in synaptic terminals of prefrontal and frontal cortex. Mol Psychiatry. 2014;19:433–43.

7. Milanese M, Zappettini S, Jacchetti E, Bonifacino T, Cervetto C, Usai C, et al. In vitro activation of GAT1 transporters expressed in spinal cord gliosomes stimulates glutamate release that is abnormally elevated in the SOD1/G93A(+) mouse model of amyotrophic lateral sclerosis. J Neurochem. 2010;113:489–501.

8. Scuderi C, Bronzuoli MR, Facchinetti R, Pace L, Ferraro L, Broad KD, et al. Ultramicronized palmitoylethanolamide rescues learning and memory impairments in a triple transgenic mouse model of Alzheimer’s disease by exerting anti-inflammatory and neuroprotective effects. Transl Psychiatry. 2018;8:32.

9. Fleck MW, Barrionuevo G, Palmer AM. Synaptosomal and vesicular accumulation of L-glutamate, L-aspartate and D-aspartate. Neurochem Int. 2001;39:217–25.

10. Bonifacino T, Musazzi L, Milanese M, Seguini M, Marte A, Gallia E, et al. Altered mechanisms underlying the abnormal glutamate release in amyotrophic lateral sclerosis at a pre-symptomatic stage of the disease. Neurobiol Dis. 2016;95:122–33.

11. Bonini D, Mora C, Tornese P, Sala N, Filippini A, la Via L, et al. Acute Footshock Stress Induces Time-Dependent Modifications of AMPA/NMDA Protein Expression and AMPA Phosphorylation. Neural Plast. 2016;2016:7267865.

12. Schneider CA, Rasband WS, Eliceiri KW. NIH Image to ImageJ: 25 years of image analysis. Nat Methods. 2012;9(7):671-5.

13. Mingardi J, la Via L, Tornese P, Carini G, Trontti K, Seguini M, et al. miR-9-5p is involved in the rescue of stress-dependent dendritic shortening of hippocampal pyramidal neurons induced by acute antidepressant treatment with ketamine. Neurobiol Stress. 2021;15:100381.

14. Preibisch S, Saalfeld S, Tomancak P. Globally optimal stitching of tiled 3D microscopic image acquisitions. Bioinformatics. 2009;25:1463–65.

15. Longair MH, Baker DA, Armstrong JD. Simple Neurite Tracer: open source software for reconstruction, visualization and  analysis of neuronal processes. Bioinformatics. 2011;27:2453–54.

16. Facchinetti R, Valenza M, Bronzuoli MR, Menegoni G, Ratano P, Steardo L, et al. Looking for a Treatment for the Early Stage of Alzheimer’s Disease: Preclinical Evidence with Co-Ultramicronized Palmitoylethanolamide and Luteolin. Int J Mol Sci. 2020;21:3802.

17. Facchinetti R, Valenza M, Gomiero C, Mancini GF, Steardo L, Campolongo P, et al. Co-Ultramicronized Palmitoylethanolamide/Luteolin Restores Oligodendrocyte Homeostasis via Peroxisome Proliferator-Activated Receptor-α in an In Vitro Model of Alzheimer’s Disease. Biomedicines. 2022;10:1236.
